# Deploying next-generation sequencing in a hospital setting

**DOI:** 10.1186/1753-6561-6-S6-P18

**Published:** 2012-10-01

**Authors:** Lars GT Jorgensen, Katherine Siminovitch

**Affiliations:** 1 Mount Sinai Hospital, Toronto, ON, Canada M5G 1X5

## 

Personalized medicine is the ability to tailor healthcare decisions based on an individual's unique characteristics (genetics, demographic information, healthcare experience, environment and social factors) to more accurately diagnose the individual's disease, predict its outcomes, and select treatments that increase the chances of a successful outcome and reduce possible adverse reactions. Moreover, it is the ability to predict an individual's susceptibility to diseases with the goal of taking measures to prevent or mitigate the extent to which an individual will experience a disease. The promise of personalized medicine in healthcare delivery includes:

• avoiding the cost of a drug or therapy with little chance of success for each patient

• avoiding exposing patients to side-effects from the wrong treatment

• avoiding increased disease burden from delays in finding the right treatment

• being able to act preventatively in cases where we can predict disease susceptibility, to delay onset and/or progression.

These changes in healthcare delivery promise improved patient outcomes as well as reduced healthcare and societal costs of managing disease.

A key component in personalized medicine is the emergence of whole-genome-scale sequencing as a platform to identify gene variants. Many challenges exist, however, to the use of large-scale sequencing in disease gene discovery and the efficacious application of such knowledge to health benefit. Leveraging upon the Ontario Government's CAD$7 million committed investment to build a clinical genomics operational framework for personalized healthcare at Mount Sinai Hospital, our project is developing a portfolio of computational tools and methods to enable acquisition, analysis, interpretation and reporting of high-throughput sequence data and the ethical, economic, psychosocial and knowledge synthesis support needed to move deep sequencing datasets and their findings into clinical practice.

The challenges to moving next-generation sequencing (NGS) into the hospital setting are both technical and regulatory. Since introduced into the research community in 2007, broad use of NGS has been greatly enabled by development of sophisticated informatics and analytic tools. While such tools have reduced many of the barriers to clinical application of NGS, the investment needed to bring NGS into medical practice remains significant, the scale of IT required being unprecedented at most hospitals. In this context, hospitals are, to some extent, at a similar place to that of research institutes when NGS first emerged. Some of the IT barriers to clinical use of NGS have been bypassed by the expertise in NGS data management developed at genome centers and across the research community. However, even this wealth of experience does not fully address the road blocks inherent to integrating NGS information into existing health workflows and IT systems, and the added challenges posed by the regulatory controls in place across the health system.

This talk will describe strategies our group is using to overcome these hurdles and thereby expedite clinical integration of NGS capability and its translation to delivery of personalized healthcare.

